# RETRACTION: Direct Formation of Hard‐Magnetic Tetrataenite in Bulk Alloy Castings

**DOI:** 10.1002/advs.202416229

**Published:** 2024-12-18

## Abstract

**RETRACTION**: Yurii P. Ivanov, Baran Sarac, Sergey V. Ketov, Jürgen Eckert, A. Lindsay Greer “Direct Formation of Hard‐Magnetic Tetrataenite in Bulk Alloy Castings,” *Advanced Science* 10, no. 1 (2023): 1–8, https://doi.org/10.1002/advs.202204315.

The above article, published online on 25 October 2023 in Wiley Online Library (wileyonlinelibrary.com) has been retracted by agreement between the journal Editor‐in‐Chief, Kirsten Severing; and Wiley‐VCH GmbH. After publication, the authors informed the journal about a potential misinterpretation of the experimental data that affected the overall conclusions of this manuscript. Subsequently, a team of authors at the University of Cambridge, including the original corresponding author, performed additional experiments which eventually confirmed that the findings presented were not correct. A Comment by this author group, that shows new experiments and discusses the reinterpretation of the original results will be published [1]. S.V.K. disagrees with the retraction while Y.P.I., B.S., J.E., and A.L.G. agree.

References

[1] Owain S. Houghton, James C. Loudon, Alison C. Twitchett‐Harrison, Nikolaos T. Panagiotopoulos, Giulio I. Lampronti, Miguel B. Costa, Richard J. Harrison, A. Lindsay Greer “Reinterpretation of Report of Tetrataenite in Bulk Alloy Castings,” *Advanced Science* 11, (2024): (accepted), https://doi.org/10.1002/advs.202408796.

**RETRACTION**: Yurii P. Ivanov, Baran Sarac, Sergey V. Ketov, Jürgen Eckert, A. Lindsay Greer “Direct Formation of Hard‐Magnetic Tetrataenite in Bulk Alloy Castings,” *Advanced Science* 10, no. 1 (2023): 1–8, https://doi.org/10.1002/advs.202204315.

The above article, published online on 25 October 2023 in Wiley Online Library (wileyonlinelibrary.com) has been retracted by agreement between the journal Editor‐in‐Chief, Kirsten Severing; and Wiley‐VCH GmbH. After publication, the authors informed the journal about a potential misinterpretation of the experimental data that affected the overall conclusions of this manuscript. Subsequently, a team of authors at the University of Cambridge, including the original corresponding author, performed additional experiments which eventually confirmed that the findings presented were not correct. A Comment by this author group, that shows new experiments and discusses the reinterpretation of the original results will be published [1]. S.V.K. disagrees with the retraction while Y.P.I., B.S., J.E., and A.L.G. agree.

**References**

[1] Owain S. Houghton, James C. Loudon, Alison C. Twitchett‐Harrison, Nikolaos T. Panagiotopoulos, Giulio I. Lampronti, Miguel B. Costa, Richard J. Harrison, A. Lindsay Greer “Reinterpretation of Report of Tetrataenite in Bulk Alloy Castings,” *Advanced Science* 11, (2024): (accepted), https://doi.org/10.1002/advs.202408796.

